# Case of Hernia Cerebri

**Published:** 1823-09

**Authors:** Wm. James Goodeve

**Affiliations:** Member of the Royal College of Surgeons, Surgeon to the Clifton Dispensary, and Lecturer on Anatomy and Surgery in Bristol.


### ART. III.—*Case of Hernia Cerebri.* By WM. JAMES GOODEVE, Esq. Member of the Royal College of Surgeons, Surgeon to the Clifton Dispensary, and Lecturer on Anatomy and Surgery in Bristol.

MAY 15th, 1820.—Evan Hopkins, æt. thirty, a quarryman, while working at St. Vincent's Rock, was struck on the forehead by a stone about the size of a man's fist, which was hurled down upon him from a height of twenty feet. He was stunned by the blow, but soon recovered sufficiently to walk a distance of nearly half a mile. I found him more than half stupefied, complaining of great pain in his head, with the scalp cut, and the frontal bone bared, but not fractured, for an extent of two inches above the left orbit. He was bled to the amount of

---

twenty ounces, and directed to take five grains of calomel, with fifteen of jalap. Wound lightly dressed.

16th.—Slept well. Complains of pain in his head; pulse quick and strong; bowels not much open.—V.S. ad ℥xx. Mist. Cathart. Emp. Lyttæ nuchæ.

17th.—Better; bowels freely open.—Rep. Mist. Cathart.

18th.—Pulse rising; pain in the head returning.—V.S. ad ℥xvj. Rep. Mist. Cathart.

19th.—Keeps his hand continually to the left side of his head; is becoming confused in his intellects; half comatose, and scarcely able to speak or swallow.—Gave an enema, which returned almost directly.

20th.—Continues in the same comatose state. Cannot get him to swallow any thing, except a spoonful of tea now and then. The slightest pressure in the vicinity of the wound gives him severe pain; the wound itself discharges particularly white good pus; he has had two very smart rigors, and these succeeded by fever, his skin being very hot and dry.

21st.—Has his hand continually on his head, as if in pain, and groans, but does not articulate; the rigors longer and more frequent; the pulse neither quick nor full. With much difficulty got him to swallow five grains of calomel.

22d.—Bowels moved, but his condition not mended; he is becoming more comatose, and his breathing difficult; he has been several times strongly convulsed; the wound is excessively tender, and rather puffy. Under these circumstances it was judged right to trephine him: I therefore enlarged the wound, found the bone apparently uninjured, (though pressure upon it gave considerable pain,) and perforated the skull. The dura mater was sound externally, and not at all detached from the skull, but puffed up as if by some effusion underneath: it was therefore divided by a small crucial incision. The opening gave vent to about a tea-spoonful of blood, and showed a quantity of serum collected under the arachnoid membrane, which was thickened and somewhat opaque. The incisions of the dura mater were enlarged as far as the margin of the trephine hole, when there immediately issued a very smart jet of arterial blood. This was allowed to flow for several minutes, and at length stopped by pressure of the finger upwards against the edge of the bone. On examining the circle of bone which was removed, I found that it was quite sound, and that it included half an inch of the left frontal sinus at its upper part. The wound was very lightly dressed with lint. The pain of the first incision roused the patient from his stupor, and he became almost ungovernable during the operation. After the operation his aspect improved, his pulse became less oppressed, and he seemed more sensible to what was passing around him.

May 23d.—Passed a tolerable night, but lies moaning and complaining of the pain on the left side of his head; the left eye is swelled; pulse 90, and of pretty good strength.—℞. Cal. gr. v.

24th.—Hemiplegia of the right side is coming on; he can scarcely open his mouth; is unable to articulate, and finds great difficulty in swallowing; has had long and severe rigors in the night; bowels open; wound discharges good pus.

25th.—Paralysis of the right side nearly complete, but he is continually throwing about the left arm and leg; the wound has a sloughy appearance; he swallows rather better to-day.—℞. Mist. Cathart.

26th.—Has been very delirious during the night, and, in spite of the hemiphlegia, contrived to get out of bed and remain in a chair for two hours; pulse 90, and not by any means strong.—℞. Mist. Cath.

27th.—Less delirious, but incessantly throwing about his left arm and leg, and gathering up the bed-clothes, but without any evident design.—℞. Cal. gr. viij.

28th.—More quiet. The calomel has procured two copious evacuations; the dura mater and subjacent parts begin to protrude through the trephine hole, above the level of the scalp; (sixth day from the operation.)

19th.—Very restless and uneasy; incessantly tossing about in the bed and picking the bed-clothes.—℞. Cal. gr. v.

30th.—The calomel has produced no evacuation from the bowels, but the patient is altogether somewhat better; the tumor has subsided a little within the trephine hole.—℞. Rep. Cal. gr. v.

31st.—The medicine has not acted on his bowels, but his gums are a little affected; he is restless, and is conscious of pain if pinched hard either in the arm or leg. There are frequent spasmodic twitchings of the muscles of the leg, which he has been observed occasionally to move very slightly.—℞. Jalapæ, gr. xv.

June 1st.—Has had one copious evacuation, and appears in every respect better; tosses less about the bed; is quite ravenous for food, though he cannot articulate; he slept well last night, but lies moaning most piteously when awake.

2d.—Moves the right leg freely; takes a good supply of jelly, milk, and panada; pulse 90, and of moderate strength; the surface of the dura mater looks sloughy.—℞. Pil. Cath. ij.

20th.—During the ensuing fortnight no material alteration took place in his general condition. His pulse ranged from 80 to 90; his appetite, though variable, was on the whole sufficiently good; the bowels were kept open by calomel and rhubarb. The paralysis of the right arm remained complete, but he could move his right leg without much difficulty; he continued unable to articulate; his urine and fæces have come away involuntarily. All this time the hernia cerebri has been gradually increasing in size, protruding through the trephine hole, and enlarging even where contiguous to the bone, so as to push up the surrounding integuments. It is now of an irregularly-rounded form and fungous appearance, two inches in height, and as much in diameter: its upper surface is divided into three distinct lobules by as many slight furrows, and these lobules have rather a sloughy appearance. The tumor is highly vascular, and has several times bled freely, but it is not by any means tender to the touch. It is kept covered with a linseed-meal poultice.

July 1st.—The tumor is evidently smaller, from sloughing of its surface. He can now pronounce Yes and No very distinctly, and has been able to move one of the fingers of his right hand. Slight tremors have been noticed in the course of the muscles of the fore-arm. He takes a good supply of broth and jelly, and is now conscious of the passage of his urine and fæces.

3d.—The sloughing of the tumor proceeds with activity, and he is more feverish and restless.

4th.—Much pain in the head, with swelling of the eyelids and integuments of the nose. There has been a considerable discharge of bloody serum from the tumor, which is on the whole rather larger just now, (about the magnitude of a middle-sized orange,) and of a soft spongy feel. Since the discharge appeared, he has been rather easier.

5th.—A large portion of the tumor, as much as would fill a dessert-spoon, has come away: its consistence is between that of a fungous tumor and coagulated blood. The man's aspect is very much mended: he looks cheerful, and is able to articulate monosyllables pretty distinctly; he can now raise his right arm and hand ten or twelve inches from the bed. The paralysis of the right side of the face remains complete.

8th.—A large breach has been made in the upper part of the tumor by sloughing, and this gives vent to a very copious thin bloody discharge. He can now utter two or three short sentences very intelligibly.

12th.—Much of the tumor has sloughed away, and he appears to be going on in every respect remarkably well; takes an adequate supply of nourishment; has a good pulse; and his bowels are sufficiently open.

14th.—Found the tumor much enlarged, and having the appearance of a spongy vascular substance, which has been bleeding copiously, and is now pulsating with violence. He has been vomiting incessantly for several hours; his face is much flushed, and his pulse 120. The vomiting is now stopped.

I know not to what cause this increased action in the tumor is to be ascribed, unless it is that his friends have been more than usually zealous in plying him with nutritious food for the last two or three days. His right arm is again powerless, and he is unable to articulate.—℞. Ol. Ricini, ℥j. statim.

15th.—The medicine has acted pretty well, but he lies in a drowsy comatose state ; the fungus appears disposed to slough. —℞. Cal. gr. v.

16th.—This fungus has now disappeared, leaving the tumor in the same state as before the formation of this last substance. His aspect is better to-day, and he has recovered the use of his right arm.

17th. There is a slight fissure around the basis of the tumor. He can now articulate more distinctly. He is allowed to take only vegetable food, and of this but sparingly. Bowels kept open by calomel and ol. ricini alternately.

21st.—The tumor remaining of the same size, with little disposition to slough, but apparently of a tougher consistence than before, I was induced to try the effect of stimulating it with red precipitate, combined with soap-cerate.

22d.—A very free discharge has taken place; the outer surface is nearly removed, and its place supplied by florid granulations. His head has been more than usually in pain.

23d.—He is restless, uncomfortable, and languid ; pulse quick; appetite not so good; the tumor quite florid, and rather larger. Seeing no good effect to result from this application, it was discontinued, and the poultice resumed.

24th.—Not so well to-day; pulse quick and weak; prostration of strength very considerable. A great deal of a brain-like substance has found its way through the hole in the centre of the tumor. Into this opening I can pass a probe to the depth of an inch below the level of the cranium, as if into the sac of an abscess, and move it about in every direction without difficulty.

25th.—Lies in a comatose state, and has frequent short rigors.

26th.—Nearly the same. There is a considerable discharge from the surface of the tumor, and from the brain itself. The probe drops by its own weight into the brain, to a depth of nearly two inches. His urine passes unconsciously.—℞. Cal. gr. v.

27th.—Free discharge from the wound ; probe sinks as far, though not quite so easily, into the brain. He is rather less comatose, and his aspect is better ; his stools are very black, and passed unconsciously.

28th.—Tumor rather smaller; suppuration free. The probe passes nearly three inches into the substance of the brain, and without producing the slightest pain.

July 31st.—Tumor lessening; suppuration free; general condition improved; suffers frequent pains in his right arm.

August 3d.—Tumor sensibly decreasing; bowels regular; tongue clean; appetite good; countenance improved; can raise his right arm, and is able to ask for food.

6th.—Has continued well until last night, when he complained of severe pain in his head, which pain has continued ever since. The bowels were freely open yesterday, and I know not to what cause this indisposition is owing, unless it is to the extreme agitation he suffered from a heavy thunder-storm last night.—℞. Cal. gr. v.

13th.—Very much better; tumor lessening; his powers of speech and motion returning.

20th.—Tumor lessening; hardly any suppuration from it.

29th.—The man is convalescent; is able to walk about, and is recovering the use of his tongue, but has a somewhat idiotic expression of countenance. Very little of the tumor is now left above the level of the trephine hole.

Sept. 10th.—No vestige of the tumor; wound completely cicatrised.

20th.—Is regaining strength, and has a less idiotic aspect.

Oct. 5th.—Suffers occasionally from slight pains in his head.

Nov. 13th.—Is able to walk as far as Clifton and back again, (a distance of three miles.) Had an epileptic fit on the 11th. —℞. Pil. Cath. iv.

Dec. 20th.—Epileptic fits recur once or twice in a fortnight, notwithstanding a very vigilant attention to the state of the bowels, and the frequent use of aperient medicine.

March 20th, 1821.—Still liable occasionally, though much less often, to epileptic fits. He has not yet lost the idiotic aspect, nor is he able to do any work.

July 15th.—Stronger and better in every respect; less liable to the fits.

August 20th, 1822.—Quite well. Has resumed his work as a labourer for some months past. He has had no return of the fits since this time twelvemonth; nor does his countenance exhibit any of that vacuity by which it was so long characterised.

*Clifton; Jan. 11th,* 1823.

